# Lessons from Drosophila: Engineering Genetic Sexing Strains with Temperature-Sensitive Lethality for Sterile Insect Technique Applications

**DOI:** 10.3390/insects12030243

**Published:** 2021-03-13

**Authors:** Thu N. M. Nguyen, Amanda Choo, Simon W. Baxter

**Affiliations:** 1 Bio21 Institute, School of BioSciences, University of Melbourne, Melbourne, VIC 3052, Australia; zoey.nguyen@student.unimelb.edu.au; 2 School of Biological Sciences, University of Adelaide, Adelaide, SA 5005, Australia; amanda.choo@adelaide.edu.au

**Keywords:** *Drosophila melanogaster*, embryo lethality, temperature sensitivity, paralysis, CRISPR/Cas9 mutagenesis

## Abstract

**Simple Summary:**

The sterile insect technique is a pest control strategy used to suppress or eliminate regional populations of insects that pose significant threats to agriculture or human health. The process involves mass-rearing, sterilization and release of male insects who fail to produce viable offspring when they mate with wild females, which leads to a population decline. Females are essential for colony propagation in rearing facilities and their selective removal prior to sterile releases remains an ongoing challenge. Developing genetic sexing strains with conditional temperature sensitive lethal mutations offers one strategy to eliminate female embryos through heat treatment, while males carry a wild type allele translocated to the Y-chromosome (or sex determination locus) to maintain their fitness. Here we review point mutations in *Drosophila melanogaster* genes that cause temperature sensitive phenotypes with the potential or ability to cause embryonic lethality. Re-engineering these known temperature sensitive mutations in other insects using CRISPR/Cas9 technology presents new opportunities to engineer genetic sexing strains for the sterile insect technique.

**Abstract:**

A major obstacle of sterile insect technique (SIT) programs is the availability of robust sex-separation systems for conditional removal of females. Sterilized male-only releases improve SIT efficiency and cost-effectiveness for agricultural pests, whereas it is critical to remove female disease-vector pests prior to release as they maintain the capacity to transmit disease. Some of the most successful Genetic Sexing Strains (GSS) reared and released for SIT control were developed for Mediterranean fruit fly (Medfly), *Ceratitis capitata*, and carry a temperature sensitive lethal (*tsl*) mutation that eliminates female but not male embryos when heat treated. The Medfly *tsl* mutation was generated by random mutagenesis and the genetic mechanism causing this valuable heat sensitive phenotype remains unknown. Conditional temperature sensitive lethal mutations have also been developed using random mutagenesis in the insect model, *Drosophila melanogaster*, and were used for some of the founding genetic research published in the fields of neuro- and developmental biology. Here we review mutations in select *D. melanogaster* genes *shibire*, *Notch*, *RNA polymerase II 215kDa*, *pale*, *transformer-2, Dsor1* and *CK2α* that cause temperature sensitive phenotypes. Precise introduction of orthologous point mutations in pest insect species with CRISPR/Cas9 genome editing technology holds potential to establish GSSs with embryonic lethality to improve and advance SIT pest control.

## 1. Introduction

### 1.1. The Sterile Insect Technique (SIT) and the Challenge of Sex Separation

The sterile insect technique (SIT) is an area-wide, environmentally friendly and species-specific biocontrol method aimed at suppressing or eliminating insect pest populations to reduce damage to crops, livestock or transmission of insect-vectored diseases. The strategy involves mass rearing and sterilization of a holometabolous pest species at the pupal stage with gamma or X-ray radiation, rendering individuals unable to produce viable offspring while maintaining the propensity to mate [1]. Sterility is caused by effects including dominant lethal chromosomal breaks and rearrangements in germ cells, which do not inhibit fertilization but do kill the embryo [2]. Periodic or sustained releases of sterilized males at densities that enable them to outcompete wild males and mate with wild females produces non-viable embryos, leading to population suppression and in some cases, eradication [3]. The SIT strategy helps reduce the use of insecticides, which can have negative environmental consequences such as chemical pollution, kill non-target beneficial organisms and result in development of insecticide resistance in wild populations [4,5]. Although SIT has been successfully applied against the New World screwworm [6], various species of fruit flies [7], moth species [8], tsetse flies [9], and mosquitoes [9,10], there are opportunities for optimisation of existing SIT programs or expansion of SIT control programs to include additional insect pest species [11,12]. The logistics of mass-rearing invasive insect pests under factory conditions for intentional release are often complex and involve many factors including strain management to maintain genetic diversity, establishing suitable larval diet for large-scale rearing, sex separation as only males are required for SIT, then effective sterilization through radiation, marking, quality control (e.g., emergence rate and flight ability), handling and distribution to obtain high-quality, cost-effective sterile males [13]. This review will focus on a major challenge facing many SIT control programs: the separation of mass-reared males from unrequired females prior to sterilization and release.

Most SIT programs lack the ability to efficiently separate mass-reared males and females thus rely on mixed-sex sterile releases. Removal of the females prior to release is highly desirable for both financial and practical reasons. Sterilized females classed as agricultural pests have the potential to cause limited damage by ‘stinging’ fruit with their ovipositor to lay ineffective embryos and this can occasionally lead to fungal, bacterial or viral infections in produce [14,15]. In the case of insect disease vector species, sterilized females might contain the potential to transmit pathogens and need to be removed before releasing. Other advantages of releasing only sterile males include enhancing the ratio of mating between sterile males and wild females [15,16], improving rapid dispersal of sterile males into native populations, and reducing the cost of mass-rearing, handling, and releasing of the insects when females are eliminated at an early developmental stage [17,18]. Insect sterilization requires optimization to achieve complete sterility, while avoiding excessive irradiation levels that may adversely damage somatic cells and impact competitiveness. Male and female insects can require different irradiation doses for sterility [19,20]. For example, Bushland [21] explained that doses of 50 gray (Gy) were required for sterilization of female screwworms, and 75 Gy was needed to completely prevent egg production, while a lower dose of 25 Gy achieved sterilization in males. Therefore, by removing females before sterilisation, the optimal radiation doses for males could be applied to improve their quality, competitiveness and to increase the effectiveness of SIT programs.

SIT has been applied to mosquito species [22,23,24] and tsetse flies [25] due to the ability to separate males and females using morphological variation and differences in developmental timing. The sex separation methods include mechanical sorting by pupal size for certain mosquito species [22] and removal of females by sorting chilled adults or based on earlier emergence times in tsetse flies [25], however, these strategies are often laborious and not easily scalable for SIT programs. Efficient genetic-based methods for sex separation of insect pest species would therefore improve the efficiency and efficacy of current SIT programs, and potentially expand implementation of SIT approaches to other pest species where it is not currently utilized.

### 1.2. Removal of Females, from Potential to Possible

Genetic sexing strains (GSS) have been developed using classical genetic approaches to enable efficient sex separation [26]. Strain development involved selection of beneficial phenotypes from polymorphic populations or through random mutagenesis, and the process has been successful for the Mediterranean fruit fly *Ceratitis capitata* [26], the Oriental fruit fly *Bactrocera dorsalis* [27], the melon fly *Zeugodacus cucurbitae* [28], the Mexican fruit fly *Anastrepha ludens* [29] and the yellow fever mosquito *Aedes aegypti* [30,31], among many others. Two principle genetic modifications are required to generate GSSs for insects with XY sex determination systems: (i) introduction of a recessive mutation that serves as a selectable marker (a visible phenotype and/or conditional lethal effect) and (ii) translocation of a wild type allele onto the male Y-chromosome, or linked to male determining loci of species with homomorphic sex chromosomes [30], to rescue functionality [26]. In the resulting strains, the females are homozygous for the mutation displaying the selectable phenotype, whereas the males are heterozygous and have a wild type phenotype.

Multiple GSSs for the Mediterranean fruit fly (Medfly), *C. capitata*, have been developed and used extensively in SIT control programs around the world. The initial Medfly GSS relied on a *white pupae* (*wp*) mutation, resulting in wild type pigmented male and white female pupae which enabled sex separation using a mechanical seed sorter [32]. This type of selectable marker however has several disadvantages: the larval diet is still required to rear females to pupation, there is up to 5% female contamination rate after sorting and pupae can be damaged by the sorting process, potentially affecting fly performance [33]. In acknowledgement of these disadvantages, a program to develop an alternative sexing system in the Medfly was initiated in 1981 at the FAO/IAEA Insect Pest Control Laboratory in Seibersdorf, Austria [33]. A suitable Medfly *temperature sensitive lethal* mutation (*tsl*) closely linked to *wp* was isolated by using mutagen ethyl methanesulfonate (EMS) and the first generation of *wp*/*tsl* GSS VIENNA-42 was made available in the early 1990s [34,35]. When heat-treating *wp*/*tsl* GSS embryos at 34 °C for 24 h, only the males survive due to the presence of a wild type copy of the *tsl-wp* genetic region translocated to the Y chromosome. This is potentially due to a protein product of a *tsl* gene functioning normally at permissive temperatures, but not at restrictive temperatures. Pupal colour provided a system to confirm the removal of females following high temperature treatment. The Medfly *tsl* has been highly successful for operational use due to the following properties: (i) removal of female embryos by heat treatment can be easily achieved using simple, accurate and inexpensive methods resulting in significant reduction of the economic costs of rearing irradiation, packaging, transport and release [33], (ii) the *tsl* mutation has no significant negative impact on the strain at normal rearing temperatures which allows for simple maintenance of the colony, and (iii) females accidentally escaping are unlikely to survive in natural environments because they are susceptible to high temperatures at all development stages [26].

**Figure 1:**
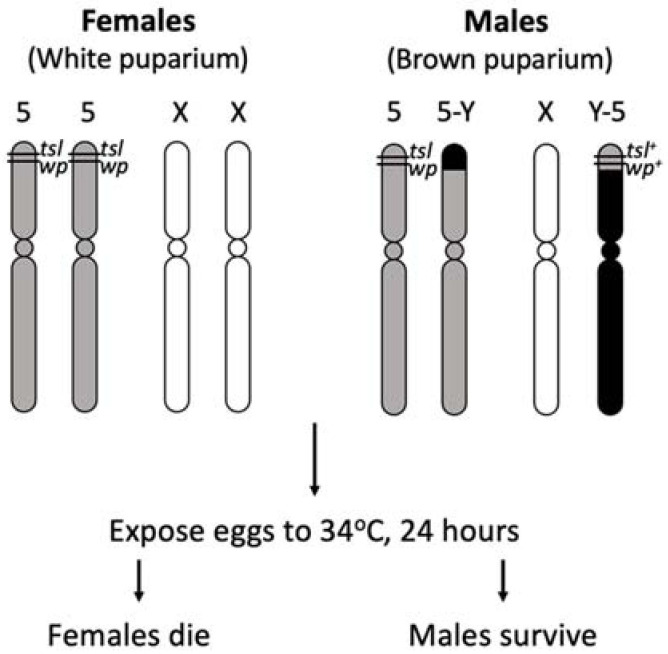
Principle chromosome structure of the Medfly VIENNA-8 GSS strain (Adapted from Franz [26]). Females are homozygous for chromosome 5 *temperature sensitive lethal* (*tsl*) and *white pupae* (*wp*) mutations and can be selectively removed with heat applications at the embryo stage. Females reared at permissive temperatures express a white pupae phenotype. Males have wild type *tsl^+^* and *wp^+^* alleles translocated to the Y chromosome resulting in resistance to temperature selection and a brown puparium.

Productivity and stability of Medfly *wp/tsl* GSSs have been associated with the positions of these causal genes relative to the Y-chromosome translocation breakpoints, and several improved strains had been developed including VIENNA-7, VIENNA-8 (Figure 1) and VIENNA-8^D53^ [17,36,37]. The stability of a GSS is threatened by rare male genetic recombination events, for example, between the wild type Y-chromosome allele and autosome carrying a mutant allele [26,38]. Recombination can result in autosomes carrying wild type alleles that gradually dilute of the ”sexing” character of the strain or produce male sensitive to temperature, which effects colony productivity. Whilst there are many advantages to the Medfly GSS, strain development has taken decades [7] and similar approaches have not produced strains for temperature sensitive sex separation in other pest species. Although the genetic basis of the Medfly *tsl* mutation is yet to be ascertained [39], a *major facilitator superfamily* gene mutation was recently found to cause the *wp* phenotype [40] and endeavours to identify alternative temperature sensitive mutations with the potential to generate GSSs for other pest species have been considered [41,42,43,44].

In addition to strategies involving classical genetics, major efforts have been undertaken to generate transgenic sexing strains (TSSs) through genetic modification. Several transgenic sexing strategies have been developed in the insect model *Drosophila melanogaster* (*D. melanogaster*) and the approaches replicated in other insect systems [45,46,47]. One common strategy involves promoters or enhancers of early-acting cellularization genes driving expression of a tetracycline-repressible transactivator gene (tTA), which in turn, drives expression of a lethal gene that is sex-specifically spliced for activity in female embryos [48,49,50], or RNAi to target female specific transcripts [51]. Rearing insects on diet containing tetracycline represses the tTA and a 1:1 sex ratio is obtained, whereas the absence of tetracycline results in ~100% female-lethality and a 50% hatching rate. The tetracycline-off system effectively removes female, yet adding antibiotic to insect diet creates additional financial costs, can impact insect viability through affecting symbiotic communities and there are environmental implications for waste disposal. Alternate transgenic sexing system have distorted sex ratios through “shredding” meiotic X-chromosomes thus favouring Y-chromosome sperm and the production of male progeny [52]. Other transgenic approaches have introduced fluorescence marker genes onto the Y chromosome or linked to a male-specific regulatory element, [53,54,55,56,57] allowing separation of transgenic males from females by automated fluorescent sorting. TSSs have multiple beneficial factors associated with them including sharing common transgene components between different insect systems, the opportunity to engineer two or more independent female elimination systems in a single strain to increase efficiency [58] and to trigger lethality or fitness costs at specific life stages [59]. Generation of TSSs requires insertion of foreign DNA from other species into the insect genome, which can result in social and legislative implications regarding their acceptance and adoption [60]. To potentially avoid these issues, Subtractive Transgene Sex Sorting has recently been developed in *D. melanogaster* as an innovative strategy to mass produce non-transgenic males by crossing specialised stocks of transgenic insects that carry tetracycline repressible lethal circuits on sex chromosomes [61].

Opportunities for developing GSSs where the female carries a conditional lethal allele exist for species with ZZ/ZW sex chromosomes, such as many Lepidoptera, where females are the heterogamic sex (ZW) [62]. Introducing a dominant conditional lethal mutation onto the female-specific W would allow both sexes to survive at permissive conditions, and select for male progeny under restrictive conditions [63]. The feasibility of this approach was demonstrated in the silkworm, *Bombyx mori*, by the stable insertion of a transgenic marker, EGFP (enhanced green fluorescent protein), into the W chromosome that could be detected at most female development stages, but not in males [64]. A dominant conditional lethal mutation inserted directly onto the W, for example using the *piggyBac* transposon [65], or translocation of a dominant gene from the Z chromosome or autosome to the W, would be required to establish a sexing strain. The development of TSSs was initiated in the codling moth, *Cydia pomonella*, [66] but low efficiency of transgenesis in this species limited the research progress [8]. Several advantages of developing strains with dominant W chromosome conditional lethal temperature sensitive mutations include: (i) elimination of females using a restrictive temperature is inexpensive (ii) insect crosses could transfer the W chromosome (carrying the dominant allele) into a genetic background that best suited a particular country or region (iii) and the released sterile males will not carry transgenic constructs or factors associated with the sexing selecting process [66].

Precise CRISPR/Cas9 genome engineering technology holds the potential to create GSSs through targeted mutagenesis, without requiring insertion of transgenes from foreign species. The CRISPR/Cas9 technology has been successfully established in many insect pest species [67,68,69,70,71] and can therefore be used to introduce *tsl* mutations into the pest genome as the first step of generating a new GSS. Whilst the genetic nature of the *tsl* mutation in the Medfly GSS is unknown, temperature sensitive mutations have been identified and characterised in other organisms including the model insect *D. melanogaster*. Establishing equivalent orthologous mutations in pest insect species using CRISPR/Cas9 has the potential to create temperature sensitive lethal strains that could be developed into GSSs. This would involve identification of suitable candidates among known *D. melanogaster* temperature sensitive mutations, introducing the orthologous mutation into the genome of the pest species using CRISPR/Cas9, and subsequently, translocation of a dominant wild type allele to the male-specific Y chromosome to rescue males, or translocation of a dominant mutant allele to the female-specific W chromosome to cause female conditional lethality.

## 2. Lessons from *Drosophila melanogaster*

The vinegar fly *D. melanogaster* was adopted as a research tool more than a century ago and is the model insect for investigating and understanding gene function. In the late 1960s and early 1970s, fundamental research into the developmental and neurobiology of multicellular organisms prompted some of the first studies in *D. melanogaster* in which temperature sensitive (*ts*) mutants were created with EMS mutagenesis [72] to help understand cell fate and regulation of behaviour [73,74,75]. Homozygous mutants exhibited wild type activity at permissive temperatures and their biological defects were examined through exposure of the insects to high temperatures during specific developmental stages. Temperature sensitive lethal (*tsl*) factors reported for *D. melanogaster* and other species such as *Saccharomyces cerevisiae* yeast [76,77], have commonly been shown to be the consequence of a single amino acid substitution in a polypeptide which alters protein activity, function or stability at different temperatures. These *tsl* mutations may have conserved function in other species, hence are potential candidates for developing GSSs. Of interest are the *D. melanogaster tsl* mutations that can satisfy three key requirements for developing GSSs: (i) they cause lethality at early development stages when embryos are treated at restrictive temperatures, (ii) females are viable and fecund at standard permissive temperatures for large scale production, and (iii) the mutation must be recessive in insects with XY/XX sex chromosomes, so female homozygotes can be efficiently eliminated and males rescued by introducing the wild type allele to the Y chromosome. Species with ZW/ZZ (female/male) sex chromosomes would benefit from dominant mutations that remove females at restrictive temperatures when inserted onto the W chromosome. The *D. melanogaster tsl* mutations discussed below are listed in Table 1.

### 2.1. Shibire

Temperature sensitive mutations in the X-chromosomal *shibire (shi)* locus were first isolated in *D. melanogaster* by Grigliatti, et al. [75]. Six different *ts* mutations (designated as *shi^ts1^* to *shi^ts6^*) that resulted in reversible adult paralysis were found by screening through 1.1 × 10^6^ progeny of EMS-mutagenized flies. Three of those mutations *(shi^ts1^*, *shi^ts3^ and shi^ts6^)* were also reported to have an embryonic lethal phenotype and larval paralysis at a restrictive temperature of 29 °C [75]. *Shi^ts2^* mutants displayed both larval and adult paralysis whereas *shi^ts4^* and *shi^ts5^* flies only showed the adult paralysis phenotype. In 1991, the *shibire* gene was found to encode *D. melanogaster* dynamin, a GTPase responsible for endocytosis and vesicle recycling [94,95]. The *D. melanogaster shi^ts1^*, *shi^ts2^* and *shi^ts4^* alleles have been reported to each contain a point mutation resulting in a single amino acid substitution (G268D, G141S and P171S, respectively) either at the boundary or within the shibire GTPase domain [95]. The homologous *shi^ts1^* mutation in human cells as well as a mutation in the *C. elegans* shibire GTPase domain have also been shown to have a temperature sensitive effect [96,97], indicating that the temperature sensitivity associated with *shibire* mutations occur in other species. Several other *shibire ts* mutants have since been reported, however not all identified at the sequence level [80,81,82,98]. One of these mutations, *shi^SHY^* (T749I), occurs within the GTPase effector domain (GED) and result in adult paralysis at 34.5 °C [80,81,82]. The majority of other mutations are dominant and result in complete homozygous lethality, with a paralytic phenotype observed in heterozygous adults [98].

In *D. melanogaster, shi^ts1^* mutant adults exhibited normal behaviour at a permissive temperature of 22 °C, whereas complete paralysis was observed within 3 min of shifting from 22 °C to 29 °C and death after exposure to 29 °C for 12–14 h [75], indicating a temperature sensitive effect. Expression of a wild type copy of *shibire* in the *shi^ts1^* mutant background was able to rescue the adult paralytic phenotype at the tested restrictive temperature of 27 °C, with *ts* induced paralysis only evident at 30–35 °C [95]. Prolonged heat treatment of 29 °C for more than 18 h at different life stages resulted in complete lethality of homozygous *shi^ts1^* embryos, larvae, pupae, and adults, which demonstrates that the *ts* mutants can be killed at higher temperatures [75,78,99]. At the permissive temperature of 22 °C, a 77% egg hatch rate, 54% larval survival and 98% prepupae eclosion was observed for homozygous *shi^ts1^* mutants. In sum, 41% of *shi^ts1^* eggs develop successfully to eclosion under permissive temperatures, suggestive of a fitness cost, however it could not be ruled out that other background mutations, or the effects of inbreeding, may also cause or contribute to high mortality rates [78].

The orthologous *shibire* gene has been identified in the Queensland fruit fly, *Bactrocera tryoni* [42], and shares approximately 90% amino acid conservation with *D. melanogaster*. CRISPR/Cas9 gene editing was used to introduce an identical *shi^ts1^* mutation into *B. tryoni* by substituting a guanine nucleotide with adenine, which altered amino acid 268 from a highly conserved glycine residue to an aspartic acid. Heterozygous *shi^ts1^* mutants were identified among G_1_ progeny and intercrossed with the aim of obtaining homozygous mutants to test for the effects of temperature sensitivity. Genetic crosses performed at a series of theoretically permissive temperatures (including 21 °C) over three generations failed to produce viable *B. tryoni shi^ts1^* homozygous mutant adults, suggestive of a homozygous lethal effect. A fitness cost associated with a single copy of the *shi^ts1^* mutant allele was also evident in heterozygotes, even at a low temperature. The observed phenotype associated with the *B. tryoni shi^ts1^* mutation was more severe than that reported for *D. melanogaster,* making it unsuitable to be used as a GSS *tsl* candidate for *B. tryoni* [42].

**Figure 2:**
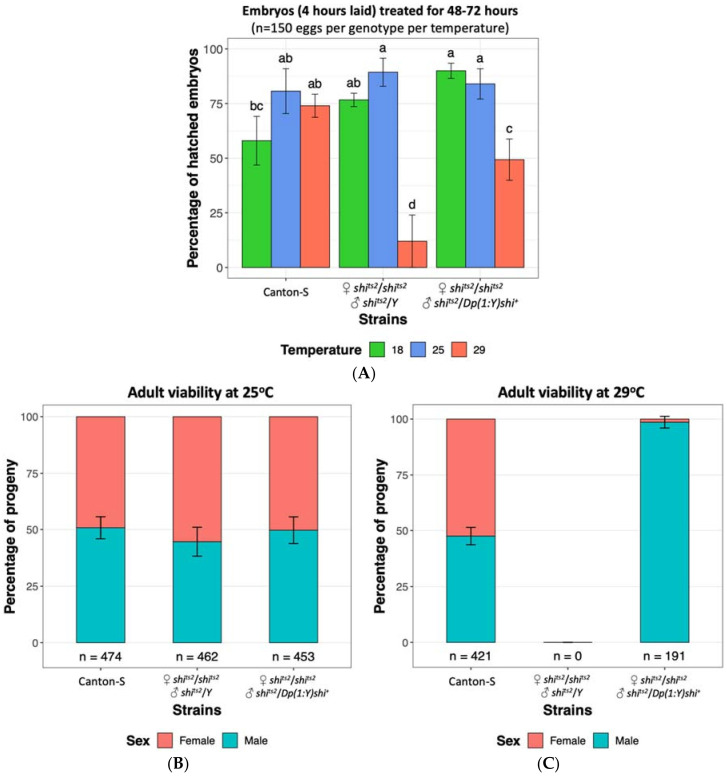
Embryo survival and fly viability at 18 °C, 25 °C and 29 °C of *Drosophila melanogaster* wild type strain Canton-S [Bloomington Stock ID 64349], temperature sensitive *shibire* strain *shi^ts2^* (♀ *shi^ts2^*/*shi^ts2^* and ♂ *shi^ts2^*/*Y*) [Bloomington Stock ID 2248] and *shibire* Y-chromosome male rescue strain (♀ *shi^ts2^*/*shi^ts2^* and ♂ *shi^ts2^*/*Dp(1:Y)shi^+^*), generated from crossing *shi^ts2^* and *Dp(1:Y)shi^+^* [Bloomington stock ID 4166]. (**A**) Embryos up to four hours old were collected and maintained at 18 °C, 25 °C and 29 °C for 48–72 h. The mean (±SD) percentage of hatched embryos from three replicates are shown, with 50 eggs per replicate. Significance was assessed using a two-way ANOVA followed by a Tukey post hoc test. The same letter above each bar indicates no significant difference (*p* > 0.05). High embryo mortality rates are observed in the *shi^ts2^* strain at 29 °C. (**B**) All strains tested are viable at 25 °C. (**C**) At 29 °C no survivorship is observed for the *shi^ts2^* strain, unless males carry the Y-chromosome rescue. Data is displayed as a percentage with the total number of flies indicated below each bar. Standard deviations from six to eight replicates are shown (Supplementary Methods S1).

The *D. melanogaster shi^ts2^* mutation has been reported to have a milder phenotype relative to *shi^ts1^* [75,78,100], with initial reports of only temporary larval and adult paralysis and no embryonic phenotype at 29 °C [75]. A more detailed examination by Poodry, et al. [78] established that *shi^ts2^* mutants displayed developmental defects upon heat treatment at 31 °C and 34 °C similar to that observed in *shi^ts1^* mutants at 29 °C. *Shi^ts1^* adults became paralysed at lower temperatures and more rapidly than *shi^ts2^* flies and conversely required more time to recover from the paralysis [100]. Although initial reports showed no evidence of the *shi^ts2^* mutation resulting in embryonic lethality, we have found that heat treatment of *shi^ts2^* embryos at 29 °C resulted in lethality of 88% of embryos compared to 26% observed for wild type Canton S flies (Figure 2A), indicating that the *shi^ts2^* mutation has a moderate effect on *D. melanogaster* embryonic viability. Whilst a low number of *shi^ts2^* adults had previously been recovered when mutants were maintained at 29 °C throughout development until prior to eclosion [78], we did not observe any adult survivors when the 29 °C heat treatment was continually applied until after the expected period of eclosion, even though *shi^ts2^* flies were viable at 25 °C (Figure 2B,C). This was supportive of the *shi^ts2^* mutation still able to result in lethality upon heat treatment albeit having a less severe effect than the *shi^ts1^* mutation. Male *shi^ts2^* mutants carrying a translocated wild type allele on the Y chromosome were protected from the heat treatment and were found to be viable at the embryonic stage and as adults (Figure 2). This observation demonstrates that the tsl effects of *shi^ts2^* can be rescued by a wild type *shibire* allele present on the Y chromosome, which is an essential criterion of a GSS.

In addition to the *D. melanogaster shi^ts2^* mutants, the *shi^ts4^* and *shi^SHY^* homozygotes also appear to have milder sensitivity to temperature compared to *shi^ts1^* homozygotes. *Shi^ts4^* adults take considerably longer to become paralysed at 29 °C compared to the *shi^ts1^* mutants [100] and Grigliatti, et al. [75] reported it as a much weaker paralytic phenotype as the adults were not entirely motionless. The *shi^ts4^* mutants were also found to be able to recover more quickly from the heat treatment. As for the *shi^SHY^* homozygotes, they were unaffected at 29 °C and had a restrictive temperature of 34.5 °C [81]. Embryonic phenotypes were not reported for either of these mutants and require investigation. The *shi^ts1^* mutation appears to have some fitness costs in *D. melanogaster* and was homozygous lethal in *B. tryoni*. Consequently, the milder *shi^ts2^*, *shi^ts4^* or *shi^SHY^* mutations may have greater potential as *tsl* candidates for generating stable GSSs.

### 2.2. Notch

The *Notch (N)* gene was cloned and sequenced in the mid-1980s, and was found to encode for a large, single heterodimeric transmembrane receptor [101,102]. The Notch protein mediates short-range signalling events and plays critical roles in cell-fate decisions both during embryonic development and in adult tissue homeostasis [103,104,105,106].

#### 2.2.1. Recessive Temperature Sensitive Lethal *Notch* Mutations

Shellenbarger and Mohler [107] isolated three recessive *tsl* alleles *N^ts1^*, *N^ts2^* and *N^Ax-ts1^* of the X-linked *Notch* locus by screening fly lines treated with the mutagen EMS. These *Notch tsl* alleles have a common lethal defect in embryonic development at restrictive temperatures above 29 °C [107]. At 18 °C, survival of homozygous *N^ts1^* and *N^ts2^* are 96% and 90%, respectively, whereas the survival of homozygous *N^Ax-ts1^* is considerably lower at only 44% [107]. Portin and Sirén [85] discovered another *Notch* recessive *tsl* allele, *N^Ax28^* induced by EMS. When reared at 25 °C, viability of homozygous and hemizygous *N^Ax28^* flies were similar to controls, whereas at 29 °C, survivorship was only 6.9%. The temperature sensitive period for *N^Ax28^* occurred the beginning of the pupal stage [85]. Both *N^Ax-ts1^* and *N^Ax28^* exhibit interrupted wing vein phenotypes at permissive and restrictive temperatures, and parallel mutations in other insect species could potentially induce similar visible traits [107,108].

The *N^ts1^* and *N^Ax28^* mutations are both single base substitutions resulting in amino acid replacements G1272D within the 32nd epidermal growth factor (EGF)-like repeat motif [109], and N986I in the 25nd EGF-like repeat motif [110], respectively. The EGF-like repeats are primary regions responsible for the interaction of Notch protein and extracellular ligands [111]. The *N^ts2^* and *N^Ax-ts1^* mutations have not been reported, however, *D. melanogaster* stocks are available (Bloomington stock ID 3075 and 3092, respectively).

Treating *D. melanogaster* homozygous and hemizygous *N^ts1^* embryos continuously at 29 °C resulted in 30% hatching rate, however, none of the hatched eggs successfully pupated even were shifted to 18 °C after hatching. Temperature sensitive periods for lethality of *N^ts1^* were localised to the first half of the embryonic period, the second and third larval instar, and to a 15-h period immediately after pupation [83].Translocation of a short X chromosome fragment carrying a wild type allele of *Notch* to the Y chromosome was able to rescue all defects of *N^ts1^* in males, including the temperature sensitive lethality at 29 °C [83].

We reassessed adult viability of *N^ts1^* (Bloomington stock ID 2533), relative to control strain *w*^1118^ (Bloomington stock ID 51629). Six replicate vials of 21 virgin flies (14 females and 7 males) were established at 25 °C for each strain and eggs were laid for 24 h. Flies were then removed and vials transferred to 18 °C or 25 °C until the progeny eclosed. The ratio of male and female progeny was expected to be relatively equal, as rearing temperatures were less than 29 °C, however, the sex ratio of *N^ts1^* flies deviated from expected at both temperatures. Male survivorship at 18 °C and 25 °C for *N^ts1^* were 36.7% (220/599) and 3.3% (8/243), respectively, whereas they were 48.4% (443/915) and 48.2% (462/959) for the control strain. This result highlights a strong fitness cost in males of this particular *N^ts1^* stock, and temperature sensitive assays comparing *Notch* wild type and *N^ts1^* alleles in the same genetic background would be useful in determining if this specific mutation is a suitable candidate for developing GSSs.

#### 2.2.2. Dominant Temperature Sensitive Lethal *Notch* Mutations

The *N^60g11^* allele was first reported by Welshons and Von Halle [86] as a dominant temperature sensitive mutation that caused a rough eye abnormality. Foster and Suzuki [112] used temperature shift experiments (21 °C to 29 °C and the reverse) to show that *N^60g11^* heterozygous females were cold sensitive during the third larval instar, which caused eye facet arrangement errors and wing nicking phenotypes. Homozygous females carrying a duplication of wild type *N* in the 2nd chromosome (*N^60g11^/N^60g11^;Dp*(1;2)51*b*) were viable when reared at 28–29 °C, whereas survival dropped to less that 3% at 20–22 °C, and the lethality was determined to occur during embryo development [87]. Fryxell and Miller [88] showed that at 26 °C, homozygous *N^60g11^/N^60g11^;Dp*(1;2)51*b* females and *N^60g11^/Y;Dp*(1;2)51*b* males had normal viability and mating success, even in competition with a wild type insect population, and rearing males at 18 °C caused lethality, indicating this *N^60g11^* phenotype was dominant over two wild type *N* alleles.

Two alterations were found in the *N^60g11^* allele [89]. The first was an amino acid substitution at codon 2257, changing a serine to glycine, which had also been reported in several other *Notch* alleles and indicated that it was not the causal variant associated with this dominant phenotype [110]. The second change was a 16 bp deletion from T15442 to A15457 inclusive, removing 5 amino acids (V2123–R2127) and introducing a frameshift resulting in translation of 19 different amino acids before termination of the protein [89]. The deletion affected cdc10-repeats, resulting in the removal of most of the Notch intracellular domain. Thus, *N^60g11^* is dominant and sensitive to cold temperature, which could be suited for genetic sexing in ZW/ZZ insects, if this allele can be inserted and effectively expressed on the female W chromosome [66].

### 2.3. RpII215

Mortin and Kaufman [113] discovered a recessive temperature sensitive mutation in the X-linked *RpII* gene encoding a subunit of RNA polymerase II which is a multi-subunit enzyme involved in catalysing the transcription of various types of RNA. The *tsl* mutation was found to affect the largest subunit that contains a molecular mass of 215,000 Daltons [114]. The single base mutation results in an amino acid substitution at codon 977 changing an arginine residue to cysteine [115], in domain 6 which forms part of the shelf module [116,117]. *RpII215^ts^* mutant flies were reported viable and fertile at the permissive temperature of 22 °C, whereas shifting of the embryos to 29 °C for 48 h caused lethality at late embryo or first instar larval stages [90,113]. Adults *RpII215^ts^* are viable if maintained at 29 °C, but the females and a proportion of the males become sterile [90,113].

**Figure 3:**
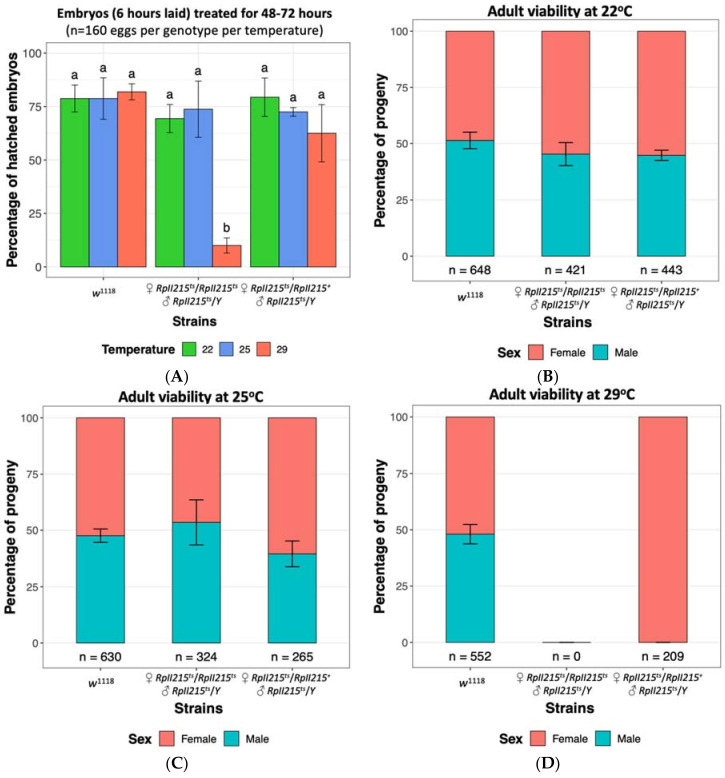
Embryo survival and fly viability at 22 °C, 25 °C and 29 °C of *Drosophila melanogaster* control strain *w*^1118^ [Bloomington Stock ID 51629], temperature sensitive *RpII215^ts^* (♀ *RpII215^ts^*/*RpII215^ts^* and ♂ *RpII215^ts^*/*Y*) [Bloomington Stock ID 34755] and their hybrid progeny (♀ *RpII215^ts^*/*RpII215^+^* and ♂ *RpII215^ts^*/*Y*). (**A**) Embryos up to six hours old were collected and maintained at three temperatures for 48–72 h. The mean (±SD) percentage of hatched embryos from four replicate are shown, with 40 eggs per replicate. Significance was assessed using a two-way ANOVA followed by a Tukey post hoc test. The same letter above each bar indicates no significant difference (*p* > 0.05). Observed female and male fly viability at (**B**) 22 °C, (**C**) 25 °C and (**D**) 29 °C temperatures confirmed rearing at 29 °C is lethal for homozygous females and hemizygous males, but not heterozygous females. Data is displayed as a percentage with the total number of flies indicated below each bar. Standard deviations from six replicates are shown (Supplementary Methods S1).

To determine if the *RpII215^ts^* strain could be reared at temperatures higher than 22 °C without fitness costs, we assessed embryo hatch rates and performed fly viability assays. There were no significant differences in hatch rates between *RpII215^ts^* mutants and *w*^1118^ control embryos at the previously reported permissive temperature of 22 °C, or when reared at 25 °C (Figure 3A). Similarly, the viability of adult mutants was unaffected at both 22 °C and 25 °C (Figure 3B,C), indicating that 25 °C is also a suitable permissive temperature for rearing *Drosophila RpII215^ts^* mutants. At the restrictive temperature of 29 °C, approximately 10% of *RpII215^ts^* embryos were able to survive the heat treatment (Figure 3A); however when the mutants were continually maintained at this high temperature throughout larval development, it resulted in complete mortality and no pupae were observed, as seen in the finding of Mortin and Kaufman [113]. Homozygous female and hemizygous male mutants failed to eclose at 29 °C, whilst heterozygous mutant females were viable, confirming that the temperature sensitive lethal phenotype for *RpII215^ts^* is recessive (Figure 3D).

### 2.4. Pale

Pendleton, et al. [91] developed a recessive temperature sensitive mutation in the *D. melanogaster pale (ple)* gene with EMS mutagenesis. The *ple* gene is located on chromosome 3 and encodes an initial rate-limiting enzyme, tyrosine hydroxylase, which is involved in the biosynthesis of 3,4-dihydroxyphenylalanine (DOPA) and dopamine [118]. Previous research has shown that tyrosine hydroxylase plays an essential role in modulating a wide range of behaviour include sleep, locomotion, courtship and learning as well as being the precursor for black melanin synthesis [118,119]. The *ple^ts^* allele is a substitution of cysteine for arginine at codon 415. Hemizygous *ple^ts1^/ple-deficiency* mutants reared throughout development at 29 °C showed almost complete lethality, had intermediate viability at 25 °C, and were viable at 18 °C [91]. Hemizygous and homozygous *ple^ts1^* mutant adults had normal locomotor activity at 18 °C, but it was significantly reduced when shifted to 25 °C and 29 °C, suggesting elevated temperatures impair enzyme function. Supplementing adult food medium with L-dopa (3 mM) successfully reversed this behavioural phenotype and restored activity to control levels [91]. Liu, et al. [120] were able to rear homozygous *ple^ts1^* flies at 25 °C, and noted that males developed notably lighter body pigmentation when moved to restrictive 31 °C temperatures after eclosion.

Pendleton, et al. [91] writes that “temperature sensitive loss of function mutant alleles of *ple* are recessive embryonic lethals” and therefore hold potential for creating orthologous conditional tsl mutations for establishing genetic sexing strains among other insect species. While homozygous *pale* knock-outs are known to be embryonic lethal [118,121], and Pendleton, et al. [91] explains *ple^ts1^/ple^ts1^* are also embryonic lethal at elevated temperatures, this evidence is based on counting flies that completed development rather than direct assessment of embryo survivorship at 29 °C. Confirming *ple^ts1^* homozygous embryos fail to hatch at restrictive temperatures would be useful to confirm death does indeed occur at this developmental stage and not as larvae or pupae. The ability to restore wild type locomotor behaviour to *ple^ts1^* homozygotes using dietary supplements of L-dopa may present useful opportunities to improve fitness of GSS for colony maintenance.

### 2.5. Transformer-2

Mechanisms for initiating sex determination among insects are diverse and can evolve rapidly [122]. Feminisation requires expression of transformer and transformer-2 proteins [123] to enable female specific splicing of doublesex pre-mRNA transcripts [124,125,126] in *D. melanogaster* and many insect species. RNAi knockdown of *tra-2* during early embryogenesis in several tephritid species, including *B. dorsalis* [127], *Bactrocera tau* [128], *C. capitata* [129] and *Anastrepha suspensa* [130], causes female to male sex conversion. Conditional conversion of XX females to males would potentially benefit SIT by doubling the number of male progeny produced from the parental population, provided all XX pseudo-males display wild type mating behaviour and the strain can be effectively propagated under permissive conditions.

Chemical mutagenesis using EMS in *D. melanogaster* generated two temperature sensitive alleles of *transformer-2* (*tra-2^ts1^* and *tra-2^ts2^*) which played an important role in demonstrating that *tra-2* expression was required throughout development to complete correct anatomical morphology and for fertility [131,132]. At 16 °C, both homozygous mutants produced normal male and female phenotypes, yet the two temperature sensitive alleles did show different severity among their phenotypes. At 16 °C *tra-2^ts1^* flies were sterile and *tra-2^ts2^* fertile, and constant rearing at 18 °C also caused sterility in *tra-2^ts2^* homozygotes. Increasing rearing conditions to 29 °C appeared to cause structural instability of both *tra-2^ts1^* and *tra-2^ts2^* protein. For example, temperature shift experiments that involved transferring homozygous *tra-2^ts1^* females from 16 °C to 29 °C twelve hours after puparium formation, caused sex-combs to develop with male morphology [131]. Shifting pupae from 29 °C to 16 °C resulted in female sex-comb morphology but male bristle number. Nevertheless, rearing XX females at 29 °C produced flies with intersex or pseudo-male phenotypes [133]. Expression of *tra-2* indirectly regulates Yolk protein (*Yp*) synthesis in female *D. melanogaster* [134], and instability of the protein may affect fertility.

Non-synonymous point-mutations were responsible for these two temperature sensitive alleles; *tra-2^ts1^* was caused by an alanine to valine substitution (GCC > GTC) at amino acid 151, and *tra-2^ts2^* caused by a proline to serine substitution (CCA > TCA) in at amino acid 181 [133]. Although the *tra-2^ts1^* allele substituted similar hydrophobic amino acids, the phenotype was severe and causes sterility when reared at 16 °C [133]. The wild type alanine residue was highly conserved and is potentially part of the ribonucleoprotein binding motif that interacts with pre-mRNA to affect splicing [135]. As the *tra-2^ts2^* mutant was viable at 16 °C in *D. melanogaster* and completely sterile at 29 °C, in addition to the reversion of XX-females to incomplete or pseudo-males, it was presented as a candidate temperature sensitive allele to test among other insects for the purpose of developing strains for SIT.

The *tra-2^ts2^* mutation (P181S) was established in both the spotted winged *Drosophila suzukii* [43] and *C. capitata* [41] using CRISPR/Cas9 homology directed repair, with the intention of creating strains sensitive to high temperatures, potentially with female to male sex reversion. In *D. suzukii*, fertile *tra-2^ts2^* males could be reared at 24 °C without dysmorphic testes and fertile females reared at 20 °C, which was a higher permissive temperature than *D. melanogaster* with the equivalent mutation. Despite this promising finding, the *tra-2^ts2^* strain and control strain failed to develop at 29 °C, and when reared at lower temperatures and shifted to 29 °C, the males did not attempt courtship or mating. Temperature sensitive mutants were difficult to rear above 26 °C, yet the *D. suzukii tra-2^ts2^* phenotype did show useful characteristics at this temperature. The XY males were sterile and expected to be competitive against wild males to mate with wild females, and XX females developed as incomplete pseudo-males that were sterile. The competitive ability for XX intersex individuals to mate with wild females remains unclear. Nevertheless, the lack of an ovipositor would be a major benefit of pseudo-male phenotype as they are unable to damage host plant fruits.

Aumann, et al. [41] successfully produced a *tra-2^ts2^ C. capitata* strain capable of female to male sex conversion at elevated temperatures. However, successful culturing of *C. capitata* is dependent upon rearing temperature, as wild type laboratory strains fail to produce progeny at 16 °C and production is reduced by 99% at 18 °C [41]. The use of *transformer-2* temperature sensitive mutations for creating genetic sexing strains may therefore be challenging in some insect species.

### 2.6. Dsor1

The gene *Downstream of raf1* (*Dsor1*, also known as *D-mek* [93]) is located on the *Drosophila* X chromosome and encodes a 393 (isoform-B) or 396 (isoform-A) amino acid serine/threonine protein kinase that plays a role in the Torso receptor tyrosine kinase signaling pathway [136]. The *Dsor1* gene is expressed throughout development and loss-of-function mutation *Dsor1*^LH110^ causes zygotic lethality [136]. Hsu and Perrimon [93] used P-element mediated transformation to develop *D. melanogaster* strains with *Dsor1* non-symonymous substitutions based on known recessive temperature sensitive point mutations in the serine/threonine kinase gene *cyclin-dependent protein kinase cdc2* of fission yeast, *Schizosacchaiomyces* *pombe*. Although Dsor1 and cdc2 are not orthologs, substituting a conserved proline residue at amino acid 137 in cdc2 and 209 in Dsor1 (isoform-B) for a serine residue was sufficient to cause temperature sensitive phenotypes [93,137]. Male flies carrying the *Dsor1^ts1^* allele (referred to as *D-mek^ts1-6^* by Hsu and Perrimon [93]) in the null lethal *Dsor1^LH110^* background could develop and were viable at both 18 °C and 20 °C (male genotype *Dsor1^LH110^*/Y; *Dsor1^ts1^*/+) but did not survive when continuously reared at 25 °C [93].

The *Dsor1^ts1^* mutation caused a range of developmental phenotypes at different temperatures. For example, the egg chorion of *Dsor1^ts1^* embryos laid at 18 °C show a weakly ventralised eggshell with fused normal length dorsal appendages, which fused and shortened at 25 °C, and reduced to knob like structures at 29 °C [93]. *Dsor1^ts1^* larvae exposed to intermittent or continuous elevated temperatures between 20 °C and 25 °C also developed a range of rough-eye phenotypes [93]. Hsu and Perrimon [93] demonstrated the Dsor1^ts1^ mutation produces a recessive temperature sensitive lethal phenotype above 25 °C. Although the precise developmental stage at which temperature sensitive lethality occurs, the mutation shows potential to be integrated into a GSS.

### 2.7. CK2α

Casein kinase 2 (CK2) is a highly conserved eukaryotic protein kinase involved in several processes essential for cellular developmental, including cell cycle regulation, cell signalling and embryogenesis, and is comprised of catalytic (CK2α) and regulatory (CK2β) subunits [138]. Recessive temperature sensitive mutations were developed in the *Saccharomyces cerevisiae ckα2* gene via hydroxylamine mutagenesis, with mutants exhibiting loss-of-function phenotypes including cell cycle arrest at restrictive temperatures above 33 °C [76]. Five *ckα2* temperature sensitive alleles were generated, four of which carried two separate amino acid substitutions, whilst the fifth mutant (*ckα2-13*) had one amino acid replacement due to a single base change (Table 2). Amongst the seven different amino acid residues that were affected, substitutions at positions A190 and D225 occurred in two separate temperature sensitive alleles. These sites are invariant in humans, *Xenopus* and *Drosophila* CK2 proteins, as well as other protein kinases [76,77].

*Drosophila CK2α* temperature sensitive mutant flies have not been reported, however, the mutations corresponding to several *S. cerevisiae* temperature sensitive alleles have been engineered into *D. melanogaster CK2α* (*dCK2α*) transgenes and transformed into yeast for complementation experiments [77]. The wildtype *dCK2α* can rescue the yeast *ckα2* loss-of-function mutant phenotype, demonstrating that the *D. melanogaster* ortholog shares similar function to the yeast. Four different *dCK2α* constructs, each encoding a single amino acid substitution (YKP7-10, Table 2) were then tested and found to rescue the yeast *ckα2* loss-of-function mutant at permissive temperatures (29 °C) but not at restrictive temperatures (37 °C). Although efficacy varied, robust rescue at permissive temperatures and complete temperature sensitive growth arrest at restrictive temperatures was confirmed for two *dCK2α* alleles, YKP7 (A177T mutation) and YKP9 (D212N mutation) [77]. Expression of the YKP8 allele resulted in incomplete rescue at the permissive temperature, which suggested that the A177V mutation has a negative effect on CK2*α* function even at lower temperatures, whilst YKP10 (G89D mutation) was a weak temperature sensitive variant as it displayed incomplete growth arrest at a 37 °C.

From these studies [76,77], it is evident that the *D. melanogaster dCK2α* orthologue can rescue wild type growth in *S. cerevisiae* mutants, and the complementation assays of four *D. melanogaster* alleles were temperature dependent. The *dCK2α* A177T and D212N transgenes produced the most robust temperature sensitive growth phenotypes in yeast. In particular, the D212N mutation is of interest as this amino acid residue is close to the CK2α active site and is thought to play an important structural role in stabilizing the catalytic subunit. It appears likely that this mutation could destabilize the protein structure at high temperatures, causing temperature sensitivity [77,138]. Whilst these *dCK2α* mutations (Table 2) have not been assessed in *D. melanogaster*, the protein is required for cellular function and growth. Thus, the *dCK2α* A177T and D212N temperature sensitive mutations appear promising temperature sensitive candidates for establishing GSSs.

## 3. What’s Still to Come—Efficient Translocation Methods

Generating GSSs for SIT requires a chromosomal translocation (or insertions) to a sex chromosome, or sex determining chromosomal region. Translocation of a wild type allele to the male determining Y chromosome will render males resistant to the effects of recessive temperature sensitive alleles, while translocation of a dominant allele, such as *N^60g11^* or others [139], to the female specific W chromosome will create restrictive phenotypes for species with female heterogametic sex chromosomes. Inducing translocations through radiation is a random process and screening for genetically stable translocation lines that do not impact insect viability is time-consuming and labour-intensive [26]. The structure of the Y-autosome translocation heavily impacts the stability and productivity of the GSS through the segregation of chromosomes during male meiosis and the ability for recombination to occur in males [26]. Karyotypes of genetically unbalanced offspring, caused by chromosomal deletions or triplications, leads to lethality [26,140] and the stability of a GSS is threatened by rare male genetic recombination events either between the translocated wild type chromosome and the free autosome carrying mutant alleles [26,38]. In the case of Medfly VIENNA-8^D53^ GSS, chromosomal inversions have improved strain stability through reducing recombination events at the *tsl* locus [36], and filter rearing systems (FRS) have helped maintain colony health and provide a system for monitoring and removal of aberrant phenotypes that may otherwise accumulate during mass-rearing [141].

The emergence of CRISPR/Cas9 genome editing system could potentially be used to induce specific Y-autosome translocations in insects. The induction of several DNA double-strand breaks simultaneously can lead to complex rearrangements in a genome by joining unrelated break ends by nonhomologous end-joining (NHEJ), thus, double-strand breaks on two distinct chromosomes can lead to reciprocal translocations. Chromosome translocations have been successfully achieved via CRISPR/Cas9 in various cell lines [142,143,144] and model organisms *Caenorhabditis elegans* [145], *D. melanogaster* [146] and *Arabidopsis thaliana* [147]. Establishing insect strains with CRISPR/Cas9 site induced translocations is thought to have potential beyond these model systems, provided adequate reference genome sequences are available to aid with experimental design [146,148]. Whether developed with CRISPR/Cas9 or classical approaches, chromosomal translocations can be stabilized with inversions to minimize unwanted recombination events. However, inversions can impact strain productivity when recombination occurs through the creating imbalanced gametes which are unable to develop [30], representing yet another challenge for maintaining stable GSS colonies for SIT.

Recombination can create unwanted genotypes among GSSs, and phenotypic markers tightly linked with temperature sensitive lethal mutations will have important roles for ongoing maintenance of mass-reared colonies. The *white pupae* locus has facilitated this role in Medfly strain variants VIENNA-7 and VIENNA-8, as it is tightly linked with the (currently unknown) embryonic lethal temperature sensitive mutation used to selectively remove females. Creating GSS based on known *D. melanogaster* temperature sensitive mutations will similarly benefit from tightly linked phenotypic markers. Strategies could potentially involve introducing transgenic fluorescent marker genes to the Y-autosome translocation chromosome, or creating mutations with visible phenotypes in genes linked with the temperature sensitive mutation. Quality assessment of strain fitness, before and after translocation experiments, will be required.

## 4. Concluding Remarks

The use of male-only releases in SIT programs minimizes health (in the case of disease vector species) and economic risks (applicable for both agriculture and disease vector pests) and increases cost efficiency (cost of mass-rearing and increased field performance of sterile males). A *temperature sensitive lethal* mutation generated by random mutagenesis has been the key to success for the Medfly GSS, but strain development and optimisation has taken considerable time. The specific *tsl* mutation currently remains unknown, yet once identified, it may be possible to replicate the same genetic alteration in other species using CRISPR/Cas9 genome editing to generate male selecting strains for SIT. Here we highlighted seven *D. melanogaster* genes, *shibire*, *Notch*, *RpII215*, *pale*, *transformer-2, Dsor1 and CK2α*, which have potential to act as temperature sensitive embryonic lethal genes if parallel mutations are introduced into the genomes of other insect species. Mutations in these seven genes largely fulfil the key criteria for generating a GSS: (i) they are recessive, (ii) they result in lethality at early development stages at restricted temperature and (iii) they are viable at normal rearing temperatures. Precise nucleotide substitution using CRISPR/Cas9 gene editing technology, will allow for the introduction of specific point mutations into insect genomes and opportunities to determine their suitability as GSS *tsl* candidates. About 75% of disease-related genes in humans [149,150] have functional orthologs in *D. melanogaster*. This statistic provides optimism that *Drosophila* embryonic temperature sensitive lethal alleles will produce the same conserved phenotype if corresponding mutations are created in other insects.

## Figures and Tables

**Table 1:** Characterised *Drosophila melanogaster* temperature sensitive mutations with potential to cause embryonic lethality if introduced into other insect species.

Name	Symbol	Chromosomal Location ^1^	Mutation ^2^	Amino Acid Substitution	Permissive Temperature	Restrictive Temperature	Phenotype (Restrictive Temperature)	References
*shibire*	*shi*	Chromosome X 15,892,116 to 15,906,716 [+]	*shi^ts1^*	G268D	22 °C	27–29 °C	Embryonic lethal	Grigliatti, et al. [75] Poodry, et al. [78]
			*shi^ts2^*	G141S	22 °C	27–29 °C	Semi-embryonic lethal	
			*shi^ts4^*	P171S	22 °C	27–29 °C	Recessive, adult paralysis	Grigliatti, et al. [75] Kim, et al. [79]
			*shi^SHY^*	T749I	22 °C	34.5 °C	Adult paralysis	Ramaswami, et al. [80] Krishnan, et al. [81] Narayanan, et al. [82]
*Notch*	*N*	Chromosome X 3,134,870 to 3,172,221 [+]	*N^ts1^*	G1272D	18 °C	29 °C	Embryonic, larval (2nd and 3rd instars) and pupal lethality reported	Shellenbarger and Mohler [83]
			*N^ts2^*	-	18 °C	30 °C	Embryonic lethal	Shellenbarger and Mohler [83] Ge, et al. [84]
			*N^Ax-ts1^*	-	18 °C	29 °C	Embryonic lethal	Shellenbarger and Mohler [83]
			*N^Ax28^*	N986I	25 °C	29 °C	Lethal beginning at pupal stage	Portin and Sirén [85]
			*N^60g11^* (Dominant)	S2257G and 16 bp deletion removing T15442 to A15457 inclusive	Above 26 °C	Below 20 °C	Embryonic lethal, cold sensitive allele	Welshons and Von Halle [86] Foster [87] Fryxell and Miller [88] Lyman and Young [89]
*RNA polymerase II 215kD subunit*	*RpII215*	Chromosome X 11,562,800 to 11,570,326 [−]	*RpII215^ts^*	R977C	22 °C	29 °C	Semi-embryonic lethal and completely lethal if continue treat at RT until first instar larvae	Mortin and Kaufman [90]
*pale (tyrosine hydroxylase)*	*ple*	Chromosome 3L 6,713,356 to 6,719,525 [−]	*ple^ts1^*	C415R	18 °C	29 °C	Embryonic lethal	Pendleton, et al. [91]
*transformer-2*	*tra-2*	Chromosome 2R 14,602,004 to 14,604,337 [−]	*tra-2^ts1^*	A151V	-	16 °C, 29 °C	Sterility, pseudo XX males	Belote and Lucchesi [92]
			*tra-2^ts2^*	P181S	16 °C	29 °C	Sterility, pseudo XX males	
*Downstream of raf1*	*Dsor1*	Chromosome X 9,247,342 to 9,250,037 [+]	*D-mek^ts1^*	P209S	20 °C	25 °C	Lethal (unreported stage of development). Temperature sensitivity observed during oogenesis.	Hsu and Perrimon [93]

^1^ NCBI reference sequences for *Drosophila melanogaster* X, 2R and 3L chromosomes are NC_004354.4, NT_033778.4 and NT_037436.4, respectively. [+] sense DNA strand, [−] anti-sense DNA strand. ^2^ Mutations are recessive unless indicated.

**Table 2:** *Saccharomyces cerevisiae ck2α* temperature sensitive alleles and *Drosophila melanogaster dCK2α* transgenes with temperature sensitive phenotypes in yeast.

*Saccharomyces cerevisiae* Hanna, et al. [76]			*Drosophila melanogaster* Kuntamalla, et al. [77]	
*Temperature sensitive* Allele	Amino Acid Substitution	Nucleotide Substitution	*Temperature sensitive* Allele	Amino Acid Substitution
*ckα2-7*	A190T	GCG → ACG	YKP7	A177T
	T336M	ACG → ATG		
*ckα2-8*	E51K	GAA → AAA		
	G102D	GGC → GAC	YKP10	G89D
*ckα2-11*	D225N	GAC → AAC		
	E299K	GAG → AAG		
*ckα2-12*	A190V	GCG → GTG	YKP8	A177V
	H294Y	CAC → TAC		
*ckα2-13*	D225N	GAC → AAC	YKP9	D212N

## Data Availability

Methods describing data generation and analysis are available in the file Supplementary Methods S1.

## Supplementary Materials

The following are available online at https://www.mdpi.com/2075-4450/12/3/243/s1. Supplementary Methods S1. Materials and Methods describing *Drosophila* husbandry and stock maintenance.
