# Supplementary material for: Lessons from Drosophila: Engineering Genetic Sexing Strains with Temperature-Sensitive Lethality for Sterile Insect Technique Applications
Source: Insects. 2021 Mar 13;12(3):243. doi: 10.3390/insects12030243 (PMC8001749; doi:10.3390/insects12030243)
Supplement: Supplementary file 1 [file insects-12-00243-s001.pdf]

Review

Lessons from Drosophila: Engineering Genetic Sexing Strains with Temperature-Sensitive Lethality for Sterile Insect Technique Applications

Thu N. M. Nguyen [1,2], Amanda Choo [2] and Simon W. Baxter [1,*]

Materials and Methods

Drosophila melanogaster stocks

All stock were obtained from the Bloomington Drosophila Stock Centre: *Notch*^{ts1} (Stock ID: 2533), *shi*^{ts2} (Stock ID 2248), *Dp(1:Y)shi*$^{+}$ (Stock ID 4166), *RpII215*^{ts} (Stock ID 34755), Canton-S (Stock ID 64349), and *w*^{1118} (Stock ID 51629).

Husbandry

Drosophila melanogaster stocks were reared at 25°C unless indicated with 65% relative humidity and a photoperiod of 12:12 h in plastic vials containing standard food media (300 g yeast, 420 g coarse semolina, 45 g J-grade agar, 476 g molasses, 46 mL acid mix (44% propionic acid, 4.4% orthophosphoric acid) and 87.3 ml tegosept (10% methyl p-hydroxy benzoate) to 10 L with water).

Embryo viability assay

Approximately 100 virgin flies 5-10 days old were placed into a cage connected to a grape agar plate containing yeast paste. Females were allowed to lay for a four or six hours period. Eggs were then counted and transferred to Petri dishes containing standard fly medium to develop either at 18°C or 25°C in constant temperature insectary rooms, a 22°C insectary or free standing incubator set to 29°C. The number of embryos that hatched were counted after 48-72 hours.

Adult viability

Crosses were set with 10-14 virgin females and 5-7 virgin males in each vial containing standard food media and left at 25°C for 24 hours for egg laying. Flies were then removed and vials containing eggs were maintained either at 18°C, 22°C, 25°C, or 29°C until emergence, then the number of males and females were counted.

Statistical analysis

All statistical analysis was performed in RStudio (v. 1.3.1056). Embryo viability was analysed using a two-way ANOVA followed by a Tukey post hoc test with temperature and strain as two factors.
